# Hypomethylation-Associated Up-Regulation of *TCF3* Expression and Recurrence in Stage II and III Colorectal Cancer

**DOI:** 10.1371/journal.pone.0112005

**Published:** 2014-11-06

**Authors:** Chunxiang Li, Sanjun Cai, Xishan Wang, Zheng Jiang

**Affiliations:** 1 Laboratory of Medical Genetics, Harbin Medical University, Harbin, Heilongjiang, China; 2 Department of Colorectal Surgery, Fudan University Shanghai Cancer Center, Shanghai, China; 3 Department of Colorectal Cancer Surgery, The 2nd Affiliated Hospital, Harbin Medical University, Harbin, China; 4 Colorectal Cancer Institute of the Heilongjiang Academy of Medical Sciences, Harbin, China; Queen Mary Hospital, Hong Kong

## Abstract

**Background and Objectives:**

Transcription factor 3 (*TCF3*) implicates Wnt signaling pathway and regulates E-cadherin expression, which is involved in aggressiveness of tumors. This study aims to investigate the role of *TCF3* in predicting prognosis of patients with stage II and III colorectal cancer (CRC).

**Methods:**

Real-Time quantitative PCR was performed in 64 fresh CRC tissues and 6 cell lines to examine *TCF3* mRNA expression. TCF3 protein expression dynamics were detected by immunohistochemistry of 118 paraffin-embedded specimens, and the clinical significance of TCF3 was assessed by clinical correlation and Kaplan-Meier analyses. Aberrant hypomethylation of *TCF3* promoter was also investigated using bisulfite sequencing and methylation specific PCR.

**Results:**

The up-regulation of *TCF3* mRNA was frequently detected both in CRC tissues with recurrence and metastasis-derived cell lines. The expression level of TCF3 protein was significantly correlated with histological type (*P* = 0.038) and disease-free survival time (*P* = 0.002). Higher TCF3 expression indicated poor prognostic outcomes (*P*<0.05, log-rank test). Multivariate analysis also showed strong TCF3 protein expression and perineural invasion were independent adverse prognosticators in CRC (*P* = 0.010, 0.000). Moreover, it was showed that promoter hypomethylation of *TCF3* is associated with its up-expression.

**Conclusions:**

This study highlighted the prognostic value of *TCF3* in stage II and III CRC. The up-regulation of *TCF3*, which is mainly caused by promoter hypomethylation, is one of the molecular mechanisms involved in the development and progression of CRC.

## Introduction

Colorectal cancer (CRC) is one of the three leading causes of cancer-related death among worldwide [1]. Stage II and III tumors together represent approximately 70% of CRC patients [2]. Regional lymph node metastasis is one of the most powerful indicators of aggressiveness of CRCs and aids in predicting the clinical outcome. However, one third of CRC patients without histological evidence of lymph node involvement die within five years after surgery from distant metastasis or local recurrence, which suggested that the nodal status may not predict the clinical course of CRC adequately [3]. It is therefore important to identify prognostic factors predicting poor outcome and guiding therapy in stage II and III CRC.

Transcription factor 3 gene (*TCF3*) was located in chromosomal band 19p13.3. *TCF3* is a ubiquitously expressed transcription regulator and encodes two basic helix-loop-helix (HLH) transcription factors, E12 and E47 [4]. These two proteins are characterized by broad expression pattern and ability to bind DNA [5]–[8]. Several studies have reported the emerging role of *TCF3* in experimental tumors. Overexpression of TCF3 has been detected in CRC [9], prostate cancer [10], [11], gastric cancer [12] and renal cancer [13]. As a transcriptional repressor of E-cadherin, *TCF3* implicated in epithelial to mesenchymal transition and may be linked to tumor aggressiveness [14]. Increasing transcriptional activity of *TCF/β-catenin* complex are the initiating event in most colorectal sporadic tumors [15]. Although considerable studies had showed *TCF3* was a tumor promoter, its role in cancer progression remains controversial [16], [17]. Patel *et al*. provided three scenarios to demonstrate the potential *TCF3* regulated mechanisms at the molecular level [11].

The goal of this study is to assess the clinical significance of *TCF3* in human CRC, and to investigate mechanisms mediating overexpression of *TCF3*, which assist in early identifying a high-risk recurrence subset of the patients with stage II and III CRC.

## Materials and Methods

### Patients and tissue specimens

From April 2000 to November 2004, 64 fresh CRC tissues, 36 with recurrence and 28 without recurrence, were collected immediately after operation at the Fudan University Shanghai Cancer Center (Shanghai, China). Characteristics of samples were showed in Table 1. Total 118 paraffin-embedded samples, 78 with recurrence and 40 without recurrence, were collected retrospectively from archival material stored in the department of pathology at Fudan University Shanghai Cancer Center (Shanghai, China) between February 1993 and March 2004, these samples were from different patients from those used for mRNA analysis (Table 2).

**Table 1:** Characteristics of 64 patients with colorectal cancer.

Characteristic	Case without recurrence	Case with recurrence	*P* a value
	(n = 28)	(n = 36)	
Age			0.454
<80	26	31	
≥80	2	5	
Sex			0.450
Male	16	24	
Female	12	12	
Size			0.615
≤5 cm	14	21	
>5 cm	14	15	
Grading			0.889
Well	6	9	
Moderate	18	21	
Poor	4	6	
Type			0.555
Adenoma	23	27	
Adenoma, mucinous	5	9	
Location of tumor			0.597
Colon	11	11	
Rectum	17	25	
Disease-free survival			0.077
≥5-y	11	23	
<5-y	17	13	
TNM stage			0.615
II	13	14	
III	15	22	
Adjuvant chemotherapy			0.615
Yes	15	22	
No	13	14	

a *P* value are obtained from χ^2^ test.

**Table 2:** Relationships between clinicopathologic features and TCF3 expression in colorectal cancer.

Variables	N (%)	TCF3 expression		*P* a value
		Strong(n = 44)	Low(n = 74)	
Sex				0.221
Male	81(69%)	27(61%)	54(73%)	
Female	37(31%)	17(39%)	20(27%)	
Age				0.194
≥65	31(26%)	15(34%)	16(22%)	
<65	87(74%)	29(66%)	58(78%)	
Location				0.838
Rectum	82(69%)	30(68%)	52(70%)	
Colon	36(31%)	14(32%)	22(30%)	
Disease-free survival				0.002*
≥5-y	52(44%)	11(25%)	41(55%)	
<5-y	66(56%)	33(75%)	33(45%)	
Histologic type				0.038*
Non-mucin-producing cancer	104(88%)	35(80%)	69(93%)	
Mucin-producing cancer	14(12%)	9(20%)	5(7%)	
Histologic grading				0.552
Low, moderate-grade	79(67%)	31(70%)	48(65%)	
High-grade	39(33%)	13(30%)	26(35%)	
Tumor size				0.532
>5 cm	35(30%)	15(34%)	20(27%)	
≤5 cm	83(70%)	29(66%)	54(73%)	
Lymphovascular invasion				0.846
Yes	46(39%)	18(41%)	28(38%)	
No	72(61%)	26(59%)	46(62%)	
Perineural invasion				0.179
Yes	17(14%)	9(20%)	8(11%)	
No	101(86%)	35(80%)	66(89%)	
TNM stage				0.833
II	40(34%)	12(27%)	28(38%)	
III	78(66%)	32(73%)	46(62%)	
Adjuvant chemotherapy				0.833
Yes	78(66%)	32(73%)	46(62%)	
No	40(34%)	12(27%)	28(38%)	

a *P* value are obtained from χ^2^ test.

*Statistically significant, *P*<0.05.

All specimens examined were taken from vital cores of histopathologically confirmed cancers at primary surgery in patients who did not undergo any local or systemic treatment before operation. Tumor samples were reviewed by at least 2 experienced pathologists, and tumor stage was assigned on the basis of system of the International Union Against Cancer. No stage II but all stage III patients received postoperative chemotherapy, but no patient received radiotherapy.

Disease-free survival was defined as the time elapsed from the date of the initial diagnosis to the appearance of local relapse or distant metastasis. Written informed consent was obtained from all patients, and the research protocol was approved by the ethics committee at Fudan University Shanghai Cancer Center.

### Cell lines

Six human CRC cell lines were obtained from the American Type Culture Collection (ATCC, Manassas, VA). Three are primary-tumor-derived lines (SW480, Caco-2, HCT116), 2 are lymph-node-metastases-derived lines (SW620, LoVo), and 1 is abdominal dropsy-metastases-derived line (Colo205). Cell lines LoVo and Colo205 were cultured in RPMI-1640 medium, whereas Caco-2 and HCT116 were cultured in Eagle's minimum essential medium and McCoy's 5a medium modified, respectively. Both SW480 and SW620 were cultured in Leibovitz's L-15 medium. All media were supplemented with 10% fetal bovine serum (GIBCO, USA), pencillin 100 IU/ml, and streptomycin 100 µg/ml at 37 °C in a 5% CO_2_- humidified atmosphere. The method of cell culture was described previously [18].

### mRNA analysis by qPCR

Total RNA was isolated with an RNeasy Mini Kit (Qiagen GmbH, Hilden, Germany) and treated with Dnase. According to the manufacturer's instructions, cDNAs were synthesized with Oligo-dT primers (Promega, Madison, WI). The *TCF3*-specific primers used were 5′-CTCGAGAAGAACAGGCCAAG-3′ (forword) and 5′-GGGGCAGGTACTGAACACAT-3′ (reverse). Glyceraldehyde-3-phosphate dehydrogenase (*GAPDH*) served as a control for normalization of gene expression and was amplified using primers 5′-GAAGGTGAAGGTCGGAGTC-3′ (forward) and 5′-GAAGATGGTGATGGGATTTC-3′ (reverse). The qPCR were carried out using the default PCR cycle on a sequence detection system ABI Prism 7900HT (Applied Biosystems), and amplified cDNA was detected by SYBR Green I dye (Qiagen GmbH, Germany). Data were analyzed using ABI Prism 7900 SDS software (Sequence Detection System 2.0; Applied Biosystems). Ratios of the intensities of the target gene and *GAPDH* signals were used as a relative measure of the expression level of target genes. All experiments were repeated three times.

### Immunohistochemistry

Archival hematoxylin and eosin-stained slides were reviewed by 2 experienced pathologists in accordence with the 2000 World Health Organization classification. A 3-tiered histologic grading system was applied. The TNM stage was evaluated according to the 2002 International Union Against Cancer classification.

A polyclonal rabbit antihuman *TCF3* antibody (dilution 1∶400) was obtained from Abcam (Cambridge, UK). In the study, 4-µm sections from archival paraffin blocks were deparaffinized and heated twice for 10 minutes each in a microwave oven (500 W) before exposure to the first antibody. Immunoperoxidase staining was carried out using the 2-step EnVision method (DAKO, Glostrup, Denmark) according to the manufacturer's instructions and visualized with 3,3′-diaminobenzidine tetrachloride (Sigma, St Louis, MO).

Cytoplasm and nuclear staining were measured for this antibody. Positive cells were counted by 2 pathologists who were blind to clinical outcome. For clinicopathological correlation, we used a 4-tiered scoring system (negative to 3+), which took into account the percentage of positive cells and staining intensity as described previously [18]. The detailed approach was used to generate a score for each tissue core as follows: no staining or staining in <10% of tumour cells (score 0), faint/barely perceptible partial staining in >10% of tumour cells (score 1+), weak-to-moderate staining in >10% of tumour cells (score 2+), and strong staining in >10% of tumour cells (score 3+). We separately interpreted TCF3 - and 1+ as ‘low expression’ and *TCF3* 2+ and 3+ as ‘strong expression’.

### Bisulfite genomic sequencing (BGS)

Genomic DNA was isolated from tissues using the DNeasy Kit (Qiagen, Hilden, Germany) and stored at −20°C before use. DNA was modified with EZ DNA Methylation-Gold KitTM (ZYMO, Orange, CA) according to the manufacturer's instruction. Generally, 1 µl modified DNA was used in subsequent PCRs. The primers for BGS are 5′-GTATAAGGTTGAAAATTTGGGT-3′ (forward) and 5′-CTCCCTAAAATCCTAAAAATCTTA-3′ (reverse). The PCR thermocycling conditions were 1 cycle at 95°C for 15 minutes; 30 cycles at 94°C for 30 seconds, 52°C for 30 seconds, and 72°C for 30 seconds and final extension at 72°C for 7 minutes. PCR products were purified with the Gel Extraction kit (Qiagen), cloned into the pMD20-T Vector (Promega, Madison, WI, USA), and then transformed into *Escherichia coli* strain DH10B. 10–15 colonies were selected to confirm the presence and size of the cloned insert. Five positive clones for each sample were selected and amplified using the vector's universal primers P2, 5′-GTAAAACGACGGCCAGT-3′, P4, 5′-AGAGGATAACAATTTCACACAGGA-3′. After purification the sequence of PCR product is analyzed using ABI 3730 DNA Sequencer (Applied Biosystems).

### Methylation specific PCR (MSP)

The primers for the methylated sequence of TCF3 gene were 5′-AATTTTATAGGAAAAAGGCGC-3′ (forward) and 5′-AACCTCGAACGCACATACTA-3′ (reverse). For unmethylated sequence were 5′-TGGAATTTTATAGGAAAAAGCTGT-3′ (forward) and 5′-AAAAACCTCAAACACACATACTA-3′ (reverse). Qiagen HotStarTaq DNA polymerase was used for PCR. Thermocycling conditions used were 1 cycle at 95°C for 15 min; 30 cycles at 94°C for 30 s, 53°C (for M primer set) or 51°C (for U primer set) for 30 s, and 72°C for 30 s; and final extension at 72°C for 7 min. PCR products were loaded 2% agarose gels followed by staining with ethidium bromide and directly visualized under UV illumination. A sample was classified as hypermethylated when the methylation amplification product alone was observed, partially methylated when both methylated and unmethylated amplification products were seen, and unmethylated when it showed unmethylated amplification products alone, or neither amplification products were found. For the statistical analysis, the hypermethylated and partially methylated samples were considered as the methylated (M) group and compared with the unmethylated (U) group [19].

### Statistical analysis

Statistical analyses were conducted with Stata (version SE/10; StataCorp, College Station, TX). The association among categorical data was analyzed by using the χ2 test. Survival curves were generated by the Kaplan-Meier method, and univariate survival distributions were compared with the use of the log-rank test. The multivariate Cox proportional hazards model was used for detection of independent prognosticator. The 2-tailed *P* value for significance was established at 0.05.

## Results

### *TCF3* expression was up-regulated in recurrent CRC tissues and CRC- metastasis-derived cell lines

**Figure 1:**
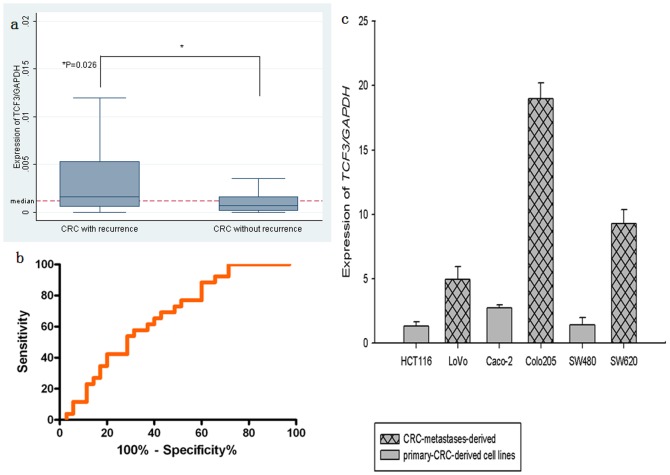
Expression of *TCF3* in 64 CRC samples. (a) In recurrent CRC, *TCF3* mRNA levels were significantly increased comared to the CRC without recurrence (Wilcoxon Signed Ranks Test, *P*<0.05). (b) ROC curve showing the performance of *TCF3* in predicting recurrence of CRC. Area under curve (AUC)  = 0.668 (95% CI 0.534–0.803), *P* value  = 0.012. (c) *TCF3* mRNA levels were measured with qPCR in 6 CRC cell lines. *GAPDH* signals were used as a relative measure of the expression level of target genes. The representative results, conducted in triplicates, are shown as mean ±SD.

In 64 fresh samples, *TCF3* mRNA expression was significantly higher in recurrent CRC tissues than in those without recurrence (*P* = 0.026; Figure 1a). Receiver-operator curve (ROC) analysis showed that the best cut-off value to distinguish between recurrent and non-recurrent CRCs was 0.0041. The the areas under the ROC curves was 0.668 (95% CI 0.534–0.803) (Figure 1b). Relative quantities of *TCF3* mRNA in CRC cell lines were expressed as N-fold difference in relation to Caco-2 and normalized to the *GAPDH* as a reference gene. The *TCF3* mRNA expression in SW620, LoVo, and Colo205 were increased 3.4-, 1.8- and 6.9-fold, respectively, compared with that of Caco-2. Whereas *TCF3* mRNA levels of SW480 and HCT116 were decreased 0.5- and 0.4-fold, respectively (Figure 1c). For 118 immunostaining samples, TCF3 protein expression was significantly associated with recurrence (Table 3).

**Table 3:** Correlation TCF3 expression with recurrence in CRC.

	Total (%)	With recurrence	Without recurrence
Strong expression	44(37%)	33(75%)	11(25%)
Low expression	74(63%)	33(45%)	41(55%)

Fisher's exact test, *P* = 0.002.

### Correlation between *TCF3* protein expression and clinicopathological features

**Figure 2:**
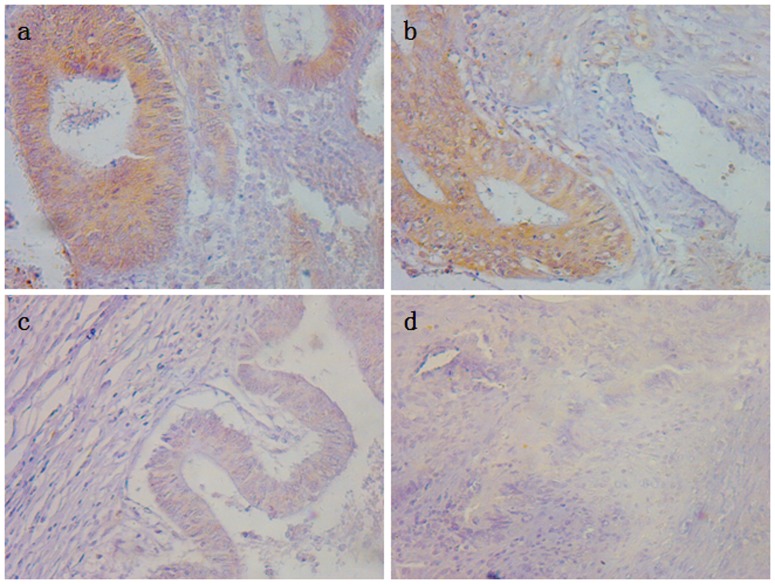
Representatives of TCF3 expression in stage II and III CRC tissues detected by immunohistochemistry. Examples of the immunostaining of TCF3 at strong expression (a, b) and low expression (c, d) levels (magnification ×400).

Positive staining was observed primarily in cytoplasm and nuclear of the cancer cells. Analysis of TCF3 expression in the 118 CRC tissues revealed that 37% of samples demonstrating strong (2+ and 3+) intensities and 63% low (− and +) intensities. Immunostaining of TCF3 protein is illustrated in Figure 2.

No significant differences were observed regarding age, sex, size, lymphvascular and perineural invasion. However, the disease-free survival rate was significantly lower in patients with high TCF3 protein expression than in those with low expression (*P* = 0.002), and TCF3 expression was significantly associated with histological type of cancer (*P* = 0.038). Table 2 showed the relations between clinicopathological features and TCF3 protein expression in118 samples.

### *TCF3* protein expression and disease-free survival time

**Figure 3:**
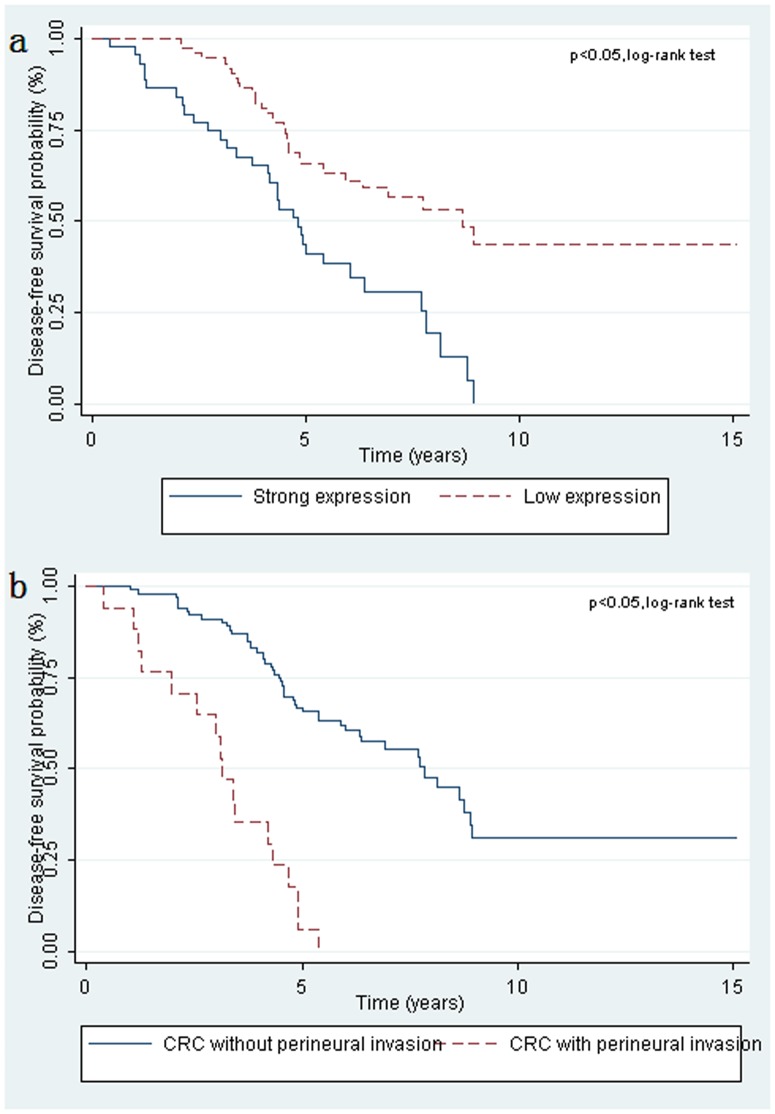
Overexpression of TCF3 was associated with a poor survival rate in CRC. (a) Patients with high TCF3 expression (n = 44) showed a significantly poorer prognisis than those with low TCF3 expression (n = 74; *P*<0.05; log-rank test). (b) Patients with perineural invasion (n = 17) showed significantly poorer prognisis than those without such invasion (n = 101; *P*<0.05; log-rank test).

Univariate analysis by Kaplan-Meier plots revealed that strong TCF3 expression was significantly associated with unfavorable disease-free survival results (*P* = 0.0001). Kaplan-Meier curves did not demonstrate any survival difference in CRC according to other parameters, including sex, age, tumor location, and lymphvascular invasion (*P*<0.05). However, survival distributions were significantly different in CRC with and without perineural invasion. Characteristic plots of TCF3 expression and perineural invasion are shown in Figure 3. Strong TCF3 expression was associated with recurrence (Table 3). Multivariate Cox analysis indicated that perineural invasion, TCF3 protein expression, and chemotherapy were independent variables (*P* = 0.00, 0.01, and 0.01) (Table 4).

**Table 4:** Univariate and multivariate analyses of disease-free survival in stage II/III CRC.

Clinical Factor	Univariate Analysis		Multivariate Analysis	
	Recurrences/No. of Patients	Log-Rank *P*	Risk Ratio (95% CI)	Wald *P*
Sex		0.74	0.979(0.561–1.709)	0.94
Female	21/37			
Male	45/81			
Age, years		0.99	0.731(0.389–1.373)	0.33
<65	49/87			
≥65	17/31			
Location		0.88	1.055(0.606–1.838)	0.85
Rectum	47/82			
Colon	19/36			
Histologic type		0.76	1.613(0.741–3.510)	0.23
Mucin-producing cancer	8/14			
Non-mucin-producing cancer	58/104			
Histologic grading		0.32	0.720(0.400–1.296)	0.27
High-grade	19/39			
Low, moderate-grade	47/79			
Tumor size		0.22	1.476(0.838–2.597)	0.18
>5 cm	22/35			
≤5 cm	44/83			
Lymphovascular invasion		0.07	1.189(0.682–2.076)	0.54
Yes	30/46			
No	36/72			
Perineural invasion		0.00*	5.031(2.508–10.092)	0.00*
Yes	17/17			
No	49/101			
Adjuvant chemotherapy		0.01*	0.5000(0.291–0.859)	0.01*
Yes	44/78			
No	22/40			
TCF3 expression		0.00*	0.493(0.288–0.844)	0.01*
Low	33/74			
High	33/44			

*Statistically significant, *P*<0.05; CI, confidence interval.

### Up-expression of *TCF3* is associated with its promoter CpG island hypomethylation

**Figure 4:**
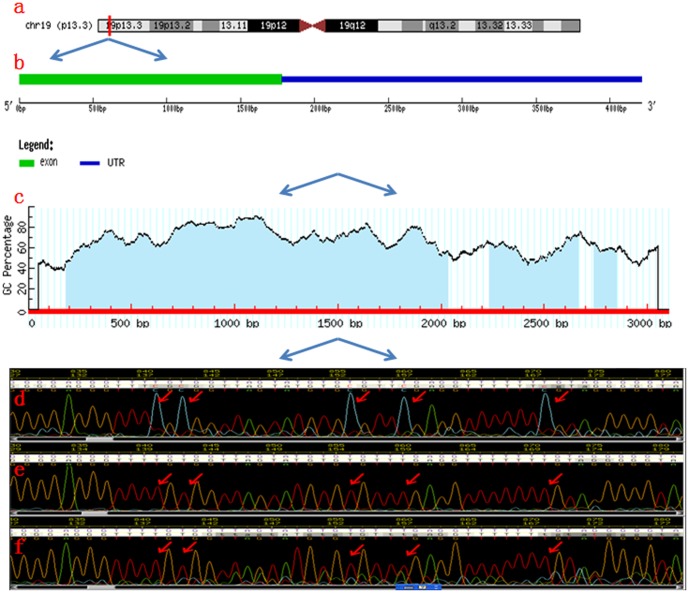
Genomic architecture of the human *TCF3* gene. Location of the *TCF3* gene within human chromosome 19 (ch19p13.3). (a) Exon structure of the human *TCF3* gene. (b) Structure of the 5′ end of the *TCF3* gene. Graph of percent guanine (G) and cytosine (C) nucleotides across this region, location of the CpG dinucleotides within this region, and boundaries of the CpG island (shaded region). (c) Representative examples of the chromatograms of CpG sites obtained from bisulphite sequencing of the *TCF3* fragment in CRC without recurrence (d) and with recurrence (e, f).

**Figure 5:**
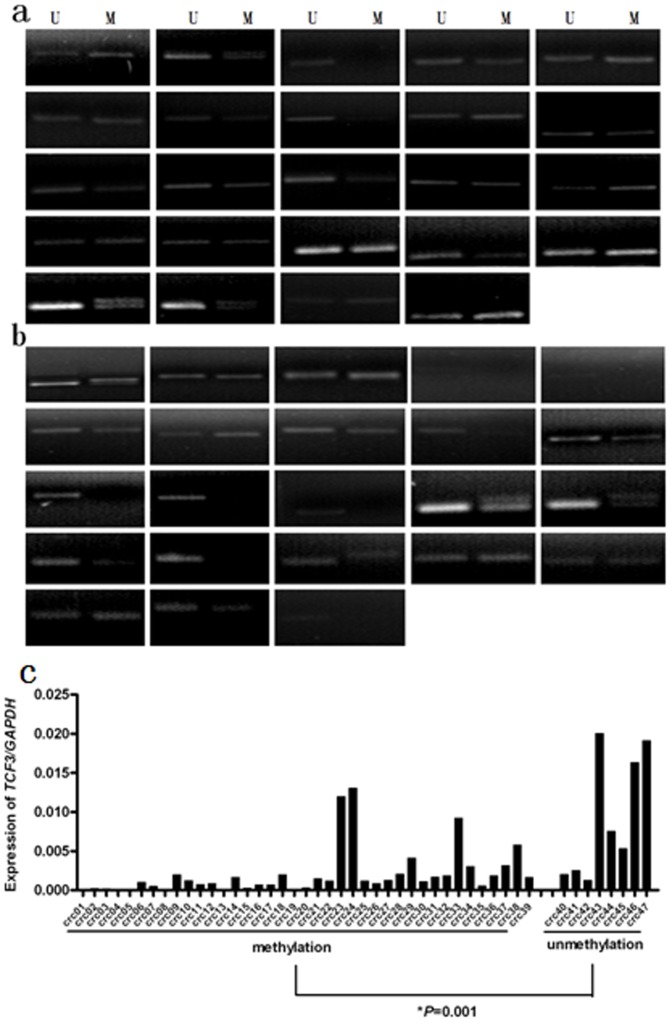
Methylation-specific PCR (MSP) analysis of *TCF3* promoter region in 24 CRC without recurrence (a) and 23 with recurrence (b). M, methylated allele; U, unmethylated allele. (c) Inverse correlation between *TCF3* promoter methylation status and gene expression level by qPCR analysis of *TCF3* expression in the 47 CRC tissues. The bar represents the ratio of *TCF3* and *GAPDH* mRNA expression levels. *TCF3* expression levels correlated with the methylation status of the gene (*P* = 0.001, Mann-Whitney U test).

A CpG island encompassing about 2.3 kb was found in the human *TCF3* gene promoter region (Figure 4c). To understand the mechanism of *TCF3* regulation in CRC, the methylation status in the promoter region of *TCF3* was tested. We amplified and sequenced the promoter region of *TCF3* using sodium bisulfite-treated genomic DNA prepared from CRC tissues. More densely methylated CpG sites (90.7% vs. 44.3%) were detected in non-recurrent CRC tissues in this study (Figure 4d, 4e, and 4f). To further validate the sequencing results, the promoter region was characterised by MSP using methylation or unmethylation-specific primers in 47 CRC specimens. Methylation of *TCF3* was detected in 23 of (95.8%) 24 non-recurrent CRC specimens. In contrast, the frequency of methylation was noticeably lower in recurrent CRC specimens (16/23, 69.6%; *P* = 0.023, Figure 5a, 5b). Furthermore, it was shown that the *TCF3* expression is significantly associated with the methylation status, indicating epigenetical inactivation might play an important role in regulatory of *TCF3* expression (*P* = 0.001, Figure 5c).

## Discussion

Our study investigates the correlation between TCF3 expression and the clinicopathological features in patients with stage II and III CRC. The TCF3 expression in recurrent CRC tissues was found significantly higher than in those without recurrence. Survival analysis showed that strong TCF3 protein expression and perineural invasion were independent adversely prognostic factors, and chemotherapy was an independent protection factor. To eliminate treatment bias caused by adjuvant chemotherapy, we analyzed the correlation between TCF3 expression and survival time in stage II and III patients, respectively. Chemotherapy did not influence the association between TCF3 expression and prognosis (Figure S1).

Hypomethylation of promoter CpG islands of oncogene is capable of activating these genes. To determine whether the upregulation of TCF3 was associated with aberrant methylation, we investigated the methylation frequency in 47 CRC tissues by MSP. The methylated allele was detected in 39 of 47 (83%) tumors tested. The frequency of methylation was significantly lower in recurrent CRC specimens compared with tumors without recurrence (*P* = 0.023). The *TCF3* expression in 39 tumors with promoter methylation was significantly lower than that in 8 cases without promoter methylation (*P* = 0.001), indicating that promoter hypomethylation was the major mechanism of the upregulation of *TCF3*. Increased *TCF/β-catenin* signaling is one of the hallmarks of CRC [20]. We, herein, provide the evidence showing methylation of *TCF3* is a common event in colorectal tumors. These data suggest that *TCF3* methylation inactivation is necessary for the inhibitory oncogenic potential of the *TCF/β-catenin* signaling. On the contrary, aberrant hypomethylation increases *TCF3* expression, which is associated with recurrence of stage II and III CRC.

The members of *TCF* family bind to unphosphorylated β-catenin [21]–[24] and regulate transcription of target genes, such as *TCF* itself and the oncogenes *cyclin D1* and *c-myc* [25], [26]. Misregulation of this signaling pathway is an important event in the development of several malignancies such as colon cancer, melanoma and prostate cancer. Besides misregulation of *TCF/β-catenin* pathway in tumor cells, it has been known that malfunction of E-cadherin allows tumor cells to invade the surrounding tissues [27]. E-cadherin expression is also reduced or absent in many epithelial cancers, including gastric and breast cancer [28]–[30]. As a transcriptional repressor, the role of *TCF3* is down-regulating E-cadherin during tumor progression [31], [32]. *TCF3* binds the E-box elements at the proximal promoter site of E-cadherin leading to transciption inactivation of E-cadherin, and the E-cadherin downregulation plays an important role in reducing cell-cell adhesion systems and promotes metastasis [33].

In view of the comprehensive data presented in this study, we propose that over-expression of TCF3 in stage II and III CRC patients was significantly associated with poor prognosis and low disease-free survival rate (Figure 3), which contributes to identify those high- risk CRC patients. Some stage II and all stage III CRC cases could be considered for adjuvant therapy [34]. However, the role of adjuvant chemotherapy in those patients with stage II tumors is still unclear [35], [36]. To select high-risk stage II disease and maximize the benefits of adjuvant therapy, an independent prognostic marker could be helpful in identifying aggressive phenotypes within stage II CRC. We have identified GAS1 as a factor to select patients with high risk of recurrence in our previous work [18], and some interesting candidate genes as prognostic factors for these samples are also validating in our laboratory (unpublished data). In this study, we showed that strong *TCF3* expression can identify a subset of patients at high-risk of recurrence and these patients may therefore benefit from more aggressive treatment.

In conclusion, our data highlights the pivotal role of *TCF3* hypomethylation in CRC recurrent development. These results underscore the potential prognostic value of *TCF3* as a biomarker for choosing adjuvant therapy for patients at high-risk of a poor outcome.

## Supporting Information

Figure S1 **Kaplan-Meier estimated survival rates according to TCF3 expression.** Patients with high TCF3 expression showed significantly poorer prognisis than those with low TCF3 expression in stage II (a) and III patients (b) (*P*<0.05, log-rank test).
(TIF)
