# Supplementary figures and images for: Hypomethylation-Associated Up-Regulation of TCF3 Expression and Recurrence in Stage II and III Colorectal Cancer
Source: PLoS One. 2014 Nov 6;9(11):e112005. doi: 10.1371/journal.pone.0112005 (PMC4222969; doi:10.1371/journal.pone.0112005)

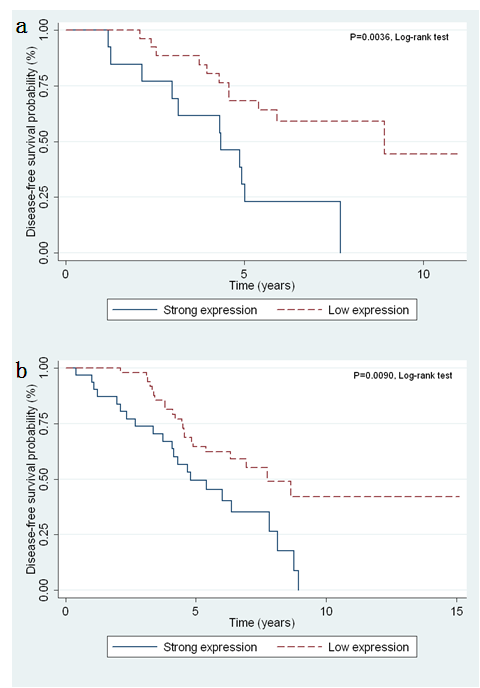

Supplement: Figure S1 — Kaplan-Meier estimated survival rates according to TCF3 expression. Patients with high TCF3 expression showed significantly poorer prognisis than those with low TCF3 expression in stage II (a) and III patients (b) (P<0.05, log-rank test). (TIF) [file pone.0112005.s001.tif]
